# Endogenous DNA Damage and Repair Enzymes —A short summary of the scientific achievements of Tomas Lindahl, Nobel Laureate in Chemistry 2015

**DOI:** 10.1016/j.gpb.2015.11.001

**Published:** 2015-12-12

**Authors:** Arne Klungland, Yun-Gui Yang

**Affiliations:** 1 Department of Microbiology, Division of Diagnostics and Intervention, Institute of Clinical Medicine, Oslo University Hospital, Rikshospitalet, Oslo NO-0027, Norway; 2 Department of Molecular Medicine, Faculty of Medicine, Institute of Basic Medical Sciences, University of Oslo, Oslo NO-0027, Norway; 3 CAS Key Laboratory of Genomic and Precision Medicine, Collaborative Innovation Center of Genetics and Development, Beijing Institute of Genomics, Chinese Academy of Sciences, Beijing 100101, China

## Abstract

Tomas Lindahl completed his medical studies at Karolinska Institute in 1970. Yet, his work has always been dedicated to unraveling fundamental mechanisms of DNA decay and DNA repair. His research is characterized with groundbreaking discoveries on the instability of our genome, the identification of novel DNA repair activities, the characterization of DNA repair pathways, and the association to diseases, throughout his 40 years of scientific career.

DNA is the genetic material that transmits all genetic information to the offspring and to do this faithfully, DNA was for long presumed to be absolutely stable. This hypothesis was challenged by the early study of Lindahl—*Rate of depurination of native DNA* [1]. He also identified numerous endogenous sources of DNA damage [2], [3], [4]. The number of DNA damages in a single human cell exceeds 10,000 every day and must be counteracted by special DNA repair processes. Tomas Lindahl summarized crucial knowledge on endogenous DNA damage and repair in an important review in 1993—*Instability and decay of the primary structure of DNA* [5]. This review also communicated fundamental knowledge on the stability of DNA to a broad audience.

Base excision repair is the repair pathway that handles most of the spontaneous lesions to our genome, such as abasic site (AP site), uracil, and various alkylated- or oxidized-DNA bases. Tomas Lindahl identified a *New class of enzymes acting on damaged DNA*, including uracil [6], [7], the DNA glycosylases. He further characterized DNA glycosylases specific for numerous damaged bases including methylated [8] and oxidized bases [9]. Furthermore, he described in detail the single-nucleotide repair patches generated following repair of uracil [10] and went on to identify all enzymes required for complete base excision repair on naked DNA and on nucleosomes [11], [12], [13], [14].

An even more sophisticated strategy for DNA repair, the adaptive response to alkylating agents, was characterized in a series of ground-breaking studies. First, he identified the methylated guanine required for the adaptive response [15] and later identified the intracellular signal [16] and the *ada* gene product with two unique functions in the induction of alkylation resistance [17]. Tomas Lindahl’s group was also the key to the identification and characterization of the AlkB family of dioxygenases [18], [19], [20]. The AlkB repair mechanism was later shown to have fundamental importance for histone demethylation, 5-methylC hydroxylation, and reversible RNA methylation.

The list of enzymes, including various DNA glycosylases, alkyltransferases, endo- and exonucleases identified and characterized by Tomas Lindahl’s group for various aspects of DNA metabolisms is nearly endless. Some examples are early studies on uracil [21], hypoxanthine [22], processing of DNA 5′ terminal ends [23], poly(ADP-ribose) [24], and the DNA ligases that complete various repair pathways by sealing nicks in DNA [26], [27], as well as more recent studies on Trex1-mediated degradation of single-stranded (ssDNA) [25]. Several mammalian repair enzymes were further characterized by the design of gene-targeted mice [28], [29], [30], [31].

It is probably less known that Tomas Lindahl, early in his scientific life, also did ground-breaking studies on the genome of the Epstein–Barr virus (EBV). Of major interest was his initial characterization of the circular EBV genome [32]. This study was followed up with a series of important publications of the EBV DNA in cancer cell lines (*e.g.*, [33]) and also included the identification of sequence variants of the Epstein–Barr genome [34].

Tomas Lindahl started his scientific career at Karolinska Institute, where he completed his PhD in 1967. He did his postdoctoral training at the Princeton University and the Rockefeller University and then became a professor at the University of Gothenburg in 1978. He is world-wide renowned also for directing the Clare Hall laboratories, part of Cancer Research UK that became a wonderful place to work and a leading center for studies on DNA repair and related processes.

On a more personal note; one, out of many, remarkable experiences working as postdocs in Tomas Lindahl’s group at Clare Hall, was his daily walks through his laboratory asking everybody “how is it going”, which could lead to a short answer or a one-hour scientific discussion. This guidance has continued for years after completing our postdoctoral training at Clare Hall, for which we are truly grateful.

Tomas Lindahl gave a keynote presentation at the “Tomas Lindahl Conference on DNA Repair” (Figure 1), organized by his two former postdocs Drs. Yun-Gui Yang and Arne Klungland in Oslo on June 20, 2015 (Figure 2).

## Figures and Tables

**Figure 1:**
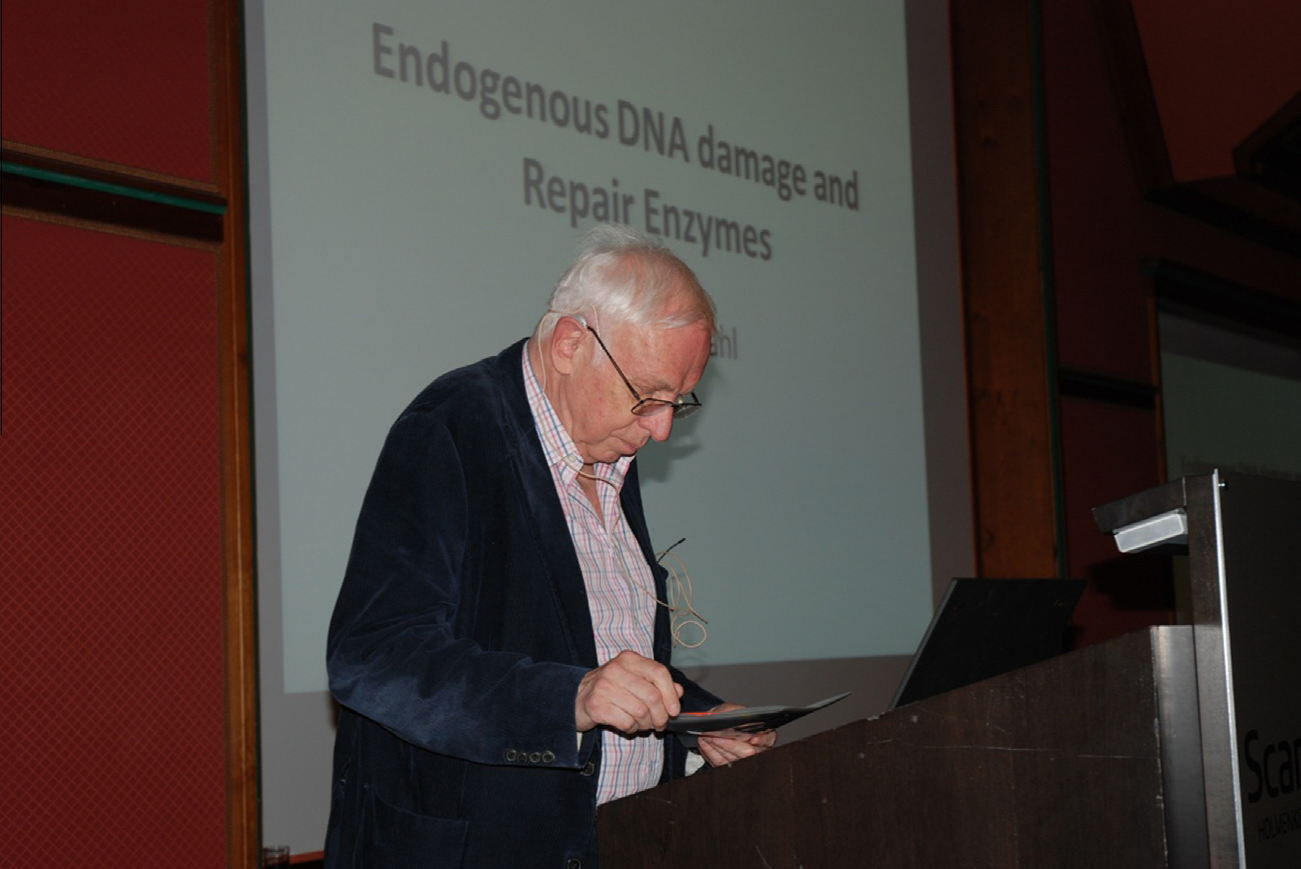
Keynote presentation by Tomas Lindahl at the “Tomas Lindahl Conference on DNA Repair”, Holmenkollen, Oslo, 2015

**Figure 2:**
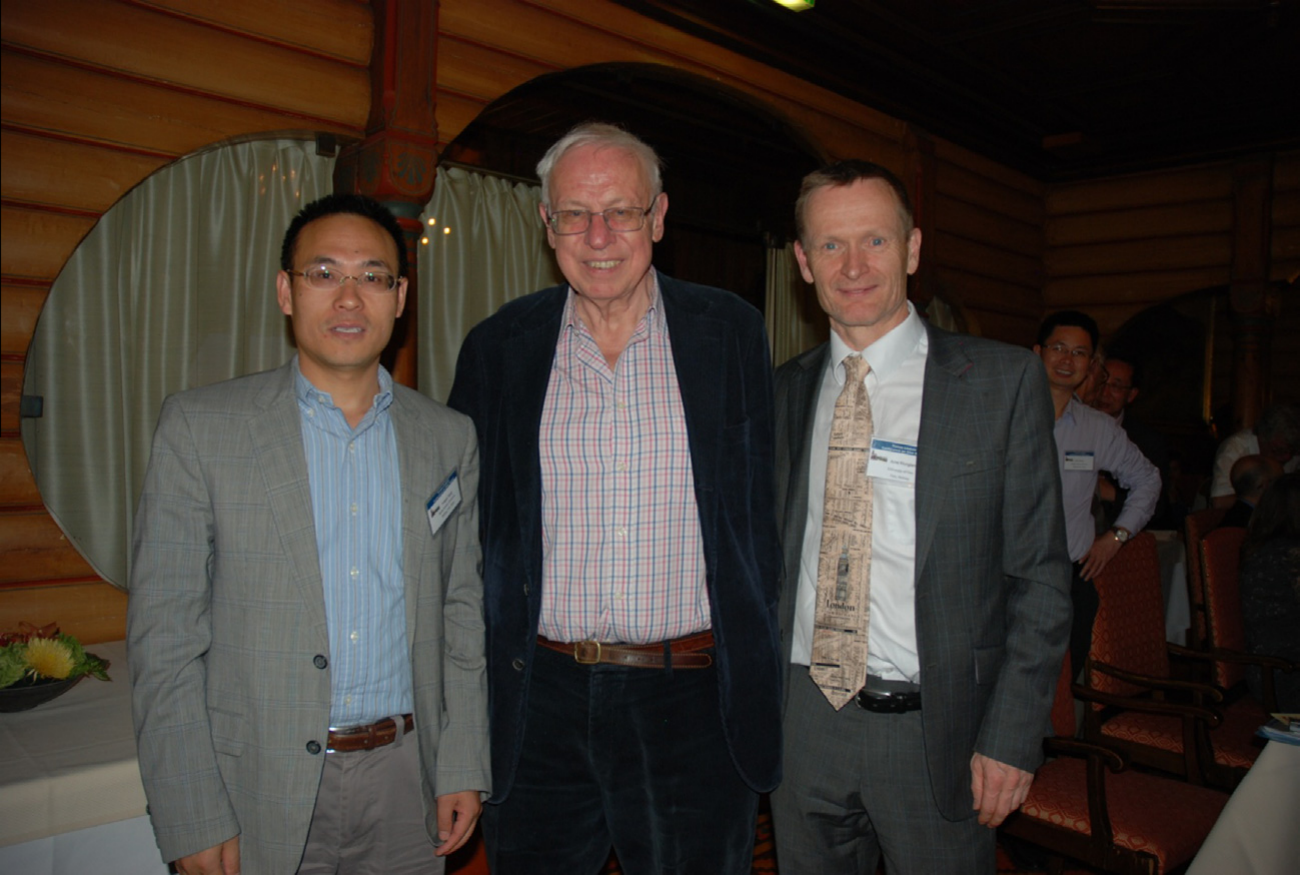
Yun-Gui Yang (left) and Arne Klungland (right) with Tomas Lindahl at the “Tomas Lindahl Conference on DNA Repair”, Holmenkollen, Oslo, 2015
